# Restrictive ventilatory impairment improved by laminoplasty for ossification of the posterior longitudinal ligament

**DOI:** 10.1002/ccr3.1959

**Published:** 2018-12-27

**Authors:** Takashi Nishida, Takashi Ishiguro, Chie Ota, Yotaro Takaku, Naho Kagiyama, Kazuyoshi Kurashima, Noboru Takayanagi

**Affiliations:** ^1^ Department of Respiratory Medicine Saitama Cardiovascular and Respiratory Center Kumagaya Saitama Japan

**Keywords:** cervical myelopathy, laminoplasty, ossification of the posterior longitudinal ligament, restrictive ventilatory impairment, type 2 respiratory failure

## Abstract

We report a woman with severe restrictive ventilatory impairment because of respiratory muscle paralysis caused by ossification of the posterior longitudinal ligament (OPLL). Laminoplasty improved her respiratory function and quality of life. Cervical myelopathy including OPLL should be considered as an important differential diagnosis in patients with respiratory dysfunction.

## 1 INTRODUCTION

Ossification of the posterior longitudinal ligament (OPLL) is a condition of abnormal calcification of the posterior longitudinal ligament and can lead to various degrees of nervous disorder resulting from compression of the spinal cord and nerve roots. OPLL can induce respiratory muscle paralysis, eventually resulting in restrictive ventilatory impairment. We report a woman with respiratory dysfunction caused by OPLL that was improved by laminoplasty.

## 2 CASE REPORT

A 67‐year‐old woman presented to our hospital with dyspnea. She developed general fatigue 5 years ago and numbness of the right body 3 years ago. She presented to an orthopedic surgeon and was diagnosed as having OPLL of the cervical spine. The neuropathy had been getting worse, and she began to feel numbness up to the extremities and had trouble walking. One year ago, she developed dyspnea on exertion. Her body weight had fallen from 46 to 41 kg over the 5 years. Spirometry conducted by a local physician revealed restrictive ventilation impairment, but the diagnosis remained unclear and she presented to our hospital for further evaluation.

**Figure 1:**
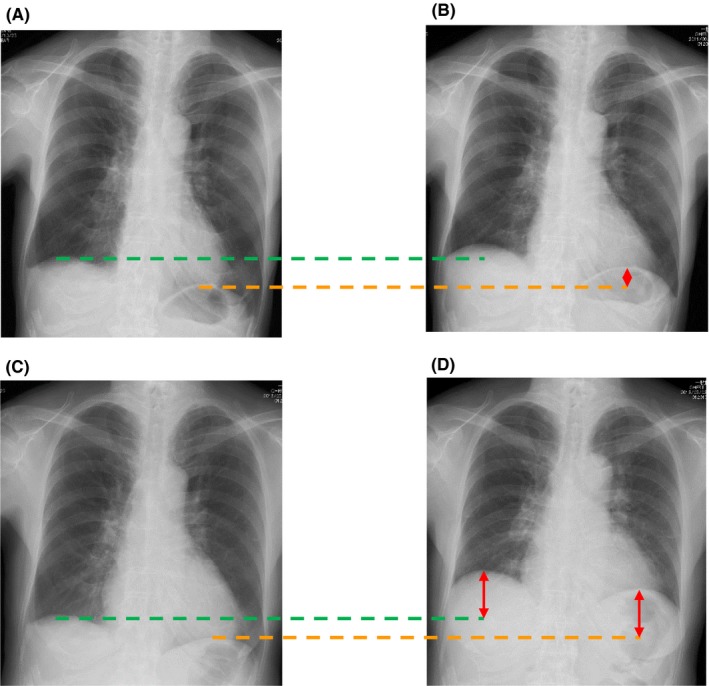
Inspiratory and expiratory chest X‐ray before and one year after laminoplasty. Preoperative inspiratory chest X‐ray did not show any abnormal shadows in both lung fields. Before laminoplasty, the height of the diaphragm of inspiration (A) and expiration (B) remains almost unchanged. One year after laminoplasty, the diaphragm of expiration (D) is lifted compared to that of inspiration (C)

**Figure 2:**
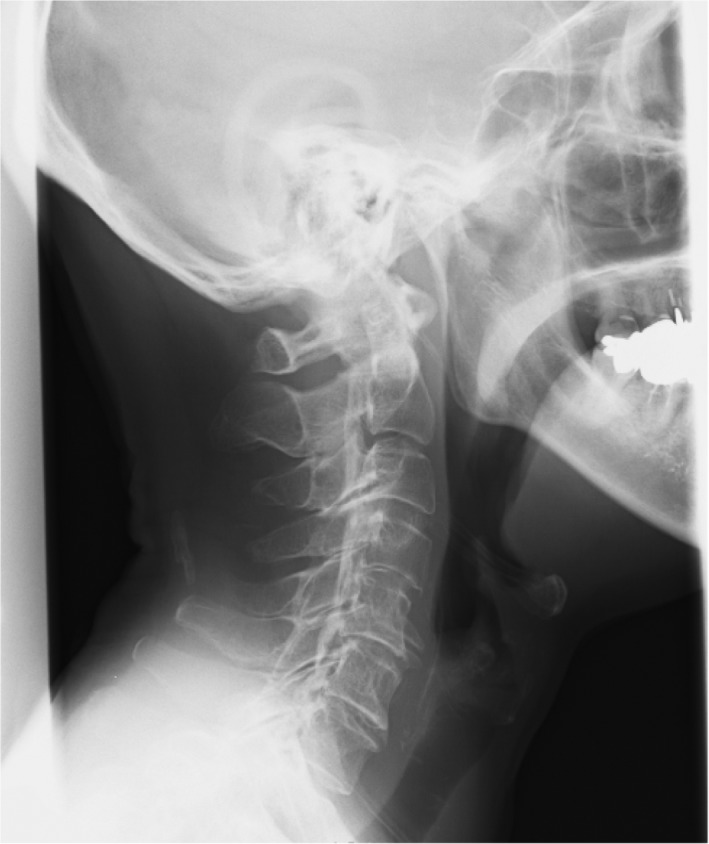
Cervical X‐ray before laminoplasty. Cervical X‐ray revealed ossification of the posterior longitudinal ligament (OPLL) of the cervical spine runs longitudinally across the vertebral body

**Figure 3:**
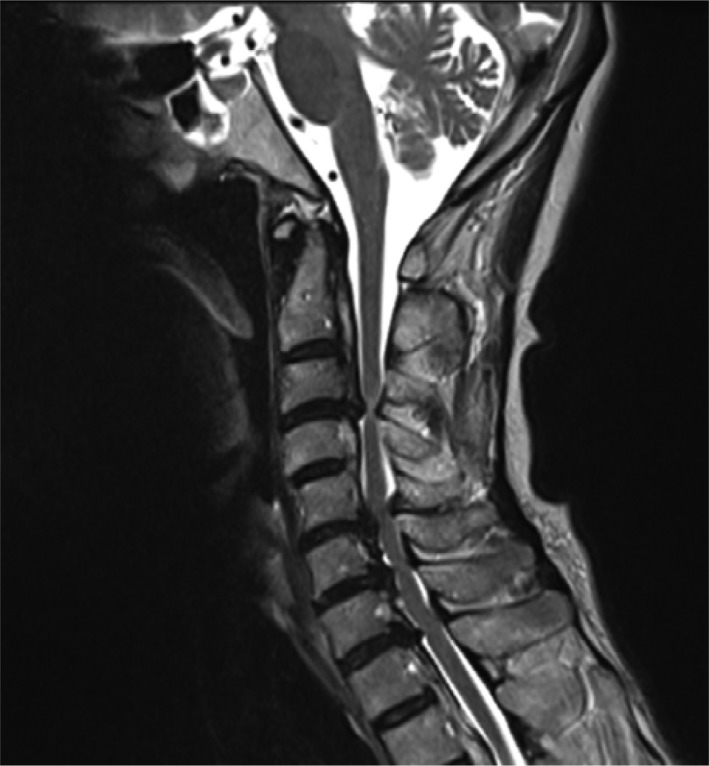
Sagittal T2‐weighted magnetic resonance image (MRI) of the cervical spine before laminoplasty. Sagittal T2‐weighted MRI revealed atrophy of the spinal cord and an intramedullary T2‐weighted high‐intensity area in the spinal cord at C3/4 level. Mild stenosis is also seen at C5/6 and C6/7, but there is no high‐intensity area

She had no medical, family, or social history of note. She had never smoked. Her vital signs included a body temperature of 36.2°C, pulse rate of 71 beats/min with a regular rhythm, and blood pressure of 103/56 mm Hg. A physical examination revealed decreased thoracic motion, muscle weakness of the right upper limb, numbness of the extremities, and claudication. No rales were audible although breath sounds were decreased in both lungs. Her Japanese Orthopaedic Association Score (JOA score)^1^ was 14 points. The JOA questionnaire grades the status of patients suffering from cervical myelopathy. A JOA score of 14 points means that cervical myelopathy is mild, and there is no indication for surgery. Chest X‐ray (Figure 1A,B) and chest computed tomography (CT) did not show any abnormal shadows in either lung field, but the movement of her diaphragm was decreased when comparing the inspiratory X‐ray with the expiratory image. Cervical X‐ray (Figure 2) revealed ossification of the posterior longitudinal ligament (OPLL) of the cervical spine runs longitudinally across the vertebral body. Sagittal T2‐weighted magnetic resonance imaging (MRI) showed a thickened posterior longitudinal ligament that was severely compressing her cervical cord at C3/4. Spinal cord MRI showed atrophic change, and an intramedullary T2‐weighted high‐intensity area was seen at the C3/4 level (Figure 3). There was no abnormality in the thoracic cord.

Her blood gasses under ambient air showed a pH of 7.36, PaO_2_ of 64.8 Torr, PaCO_2_ of 61.3 Torr, HCO_3_^‐^ of 33.6 mmol/L, and alveolar‐arterial oxygen pressure difference of 1.18 Torr. No other remarkable changes were seen in the biochemical examination of her blood and urine. Pulmonary function test results (% predicted) showed restrictive ventilatory impairment: vital capacity (VC) of 1.09 L (39.4%), FEV1 of 0.99 L (62.7%), and FEV1/FVC of 98%. Polysomnography showed obstructive sleep apnea (OSA) with an apnea‐hypopnea index (AHI) of 20.7 and minimum oxygen saturation of 86%. From these findings, we considered that cervical myelopathy due to OPLL was causing her respiratory muscle paralysis and that OSA might also be affecting its course.

First of all, we started continuous positive airway pressure (CPAP) only at night, and her AHI and minimum saturation with CPAP improved to 13.9% and 88%, respectively, but her daytime hypercapnia and dyspnea on exertion did not improve. In addition, the neuropathy of her extremities was worsening, and C3‐7 laminoplasty was performed.

**Figure 4:**
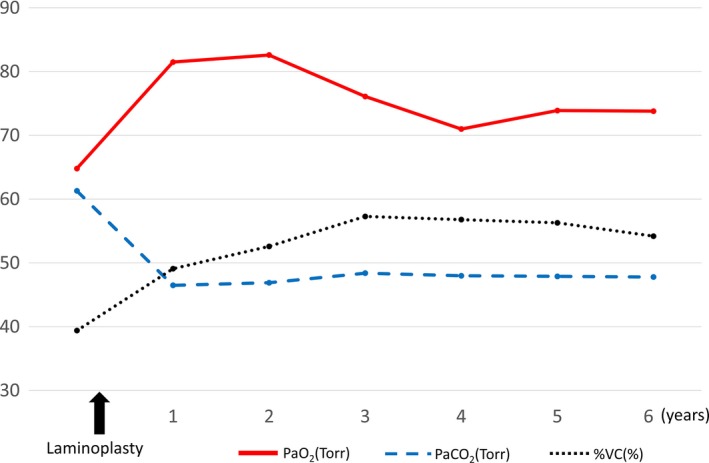
Changes in PaO_2_, PaCO_2_, and %VC. Preoperative blood gas analysis and pulmonary function test show hypoxemia, hypercapnia, and severely reduced vital capacity (VC). One year after laminoplasty, both blood gas analysis and VC improved and have been maintained without deterioration for 6 years after surgery

Although her pulmonary function did not improve immediately after surgery, it began to gradually and steadily improve over the first postoperative year. The mobility of her diaphragm increased during inspiration/expiration (Figure 1C,D), and her blood gasses also improved (Figure 4). One year after laminoplasty, her PaO_2_ improved from 64.8 to 85.4 and PaCO_2_ from 61.3 to 40.9 Torr, and they have been maintained without deterioration for 6 years after surgery. Pulmonary function testing also showed improvement in her %VC postoperatively, from 39.4% to 57.3% 3 years after surgery. Although it has declined somewhat since the 4th year, it is still maintained at a value that is 15% higher than that before surgery. Her AHI by polysomnography without CPAP also improved from 20.7 to 9.0, so the CPAP treatment was stopped. With the improved pulmonary function, her chief complaints of dyspnea on exertion and general fatigue also disappeared. She has regained her daily life and is able to exercise lightly at a fitness club.

## 3 DISCUSSION

Ossification of the posterior longitudinal ligament of the cervical spine is one of the causes of cervical myelopathy and can lead to stenosis of the cervical spinal canal, compressing the cervical spinal cord and producing various neurological disorders. Up to 25% of patients presenting with cervical myelopathy may have features of OPLL.^2^ Many patients with OPLL are found in East Asia, especially in Japan.^3^ Although its etiology has not been elucidated, some reports suggest a relationship with genetic characteristics.^4^, ^5^

Ossification of the posterior longitudinal ligament compresses the spinal cord and leads to various symptoms such as rigidity of the extremities, increasing deep tendon reflex, extremity dysesthesia, and claudication. It can also injure the nerves controlling the respiratory muscles, resulting in their paralysis and decreased pulmonary function.^6^ For example, the diaphragm, which is innervated by the phrenic nerve, is primarily responsible for ventilation and is aided by intercostal muscles. The phrenic nerve originates from the C3 to C5 nerve roots. Therefore, an upper cervical disorder tends to cause severe ventilatory impairment and hypoventilation.

Acute spinal cord injury is likely to cause respiratory dysfunction and can lead to respiratory arrest. Meanwhile, respiratory dysfunction in chronically progressive cervical myelopathy is often subtle and subclinical, and respiratory problems rarely become the main symptoms. There are several reports showing that pulmonary function (especially VC) decreases due to cervical myelopathy including OPLL, and some show improvement in pulmonary function after cervical spine surgery^7^, ^8^, ^9^, ^10^, ^11^ (Table 1). However, the preoperative %VCs of patients with chronic cervical myelopathy in these reports were as mild as 65.0%‐97.9%, whereas the %VC of our patient was reduced to 39.4%. The possible reasons in our patient are as follows: (a) severe compression of the spinal cord, (b) general muscle weakness: Long‐term neurological disorder and respiratory dysfunction caused weight loss and general muscle weakness, (c) coexisting sleep apnea, and (d) the anatomical characteristics of OPLL: Motor and sensory functions of the extremities are controlled through tracts in the posterior two thirds of the cervical spinal cord, whereas respiratory muscles are controlled through tracts in the anterior one third.^6^, ^12^, ^13^ OPLL compresses the anterior spinal cord, which might have induced stronger respiratory dysfunction relative to the degree of neurological symptoms in her extremities. In fact, one report showed that the preoperative %VC was actually lower in the OPLL group than that in the cervical spondylotic myelopathy group.^14^ There are few reports of OPLL causing symptomatic respiratory dysfunction, possibly because OPLL is underrecognized as a cause of type 2 respiratory failure and restrictive ventilatory impairment.

**Table 1:** Previous reports showing pre‐ and postoperative vital capacity (% predicted) in patients with cervical myopathy

Ref	Year	Localization	N	Pre %VC	Post %VC	*P* value
7	2001	Anywhere	12	91.2	88.7	NS
8	2002	Anywhere	52	97.9	99.3	NS
		At or above C3/4	24	92.5	97.4	0.004
9	2012	Anywhere	31	89.4	88.4	NS
		At or above C3/4	13	81.8	83.8	NS
10	2012	Anywhere	49	86.0	87.4	NS
		At or above C3/4	16	81.1	83.0	NS
11	2016	Anywhere	30	65.0	73.7	0.003

%, percent predicted; Anywhere, anywhere in the cervical spine; C, cervical vertebrae; N, number of patients; NS, not significant; Post, postoperative; Pre, preoperative; Ref, reference; VC, vital capacity.

There are many reports of OSA due to acute cervical spine injury, but few reports show an association between chronic progressive cervical spine disease and OSA. Hasegawa et al^15^ reported a case of OSA with cervical myelopathy improved by surgery. Our patient also had coexisting OSA, and CPAP was used only at night. Although CPAP improved her AHI from 20.7 to 13.9, it did not improve her daytime hypercapnia. However, at one year after surgery, her AHI on polysomnography without CPAP had improved from 20.7 to 9.0. This suggests some relation between her cervical myelopathy due to OPLL and OSA. However, the detailed mechanism for this is unknown, so we need to accumulate and analyze further cases.

In conclusion, we report a patient with OPLL in whom laminoplasty improved her respiratory dysfunction and OSA. When differentiating the cause of type 2 respiratory failure and restrictive ventilatory impairment, the possibility of both neurological disease and lung disease should be considered. Cervical myelopathy including OPLL should be considered especially in Asians because cervical spine surgery may improve pulmonary function and hypercapnia.

## CONFLICT OF INTEREST

None declared.

## AUTHOR CONTRIBUTION

TN: is the guarantor of the paper, taking responsibility for the integrity of the work as a whole, from inception to published article. TI, CO, YT, NK, KK, and NT: aggregated the data, created the tables, and helped draft the discussion of the manuscript.
